# Root phenotypes of young wheat plants grown in controlled environments show inconsistent correlation with mature root traits in the field

**DOI:** 10.1093/jxb/eraa201

**Published:** 2020-04-29

**Authors:** Sarah M Rich, Jack Christopher, Richard Richards, Michelle Watt

**Affiliations:** 1 CSIRO Agriculture and Food, Perth, WA, Australia; 2 CSIRO Agriculture and Food, Canberra ACT, Australia; 3 University of Queensland, Queensland Alliance for Agricultural and Food Innovation, Leslie Research Centre, Toowoomba, QLD, Australia; 4 School of BioSciences, University of Melbourne, Parkville, Victoria, Australia; 5 Lancaster University, UK

**Keywords:** Deep root, field to lab, lab to field, root angle, root architecture, root elongation, root number, root phenotyping, root screen, *Triticum aestivum*, wheat

## Abstract

Using a field to lab approach, mature deep-rooting traits in wheat were correlated to root phenotypes measured on young plants from controlled conditions. Mature deep-rooting root traits of 20 wheat genotypes at maturity were established via coring in three field trials across 2 years. Field traits were correlated to phenotypes expressed by the 20 genotypes after growth in four commonly used lab screens: (i) soil tubes for root emergence, elongation, length, and branching at four ages to 34 days after sowing (DAS); (ii) paper pouches 7 DAS and (iii) agar chambers for primary root (PR) number and angles at 8 DAS; and (iv) soil baskets for PR and nodal root (NR) number and angle at 42 DAS. Correlations between lab and field root traits (*r*^2^=0.45–0.73) were highly inconsistent, with many traits uncorrelated and no one lab phenotype correlating similarly across three field experiments. Phenotypes most positively associated with deep field roots were: longest PR and NR axiles from the soil tube screen at 20 DAS; and narrow PR angle and wide NR angle from soil baskets at 42 DAS. Paper and agar PR angles were positively and significantly correlated to each other, but only wide outer PRs in the paper screen correlated positively to shallower field root traits. NR phenotypes in soil baskets were not predicted by PR phenotypes in any screen, suggesting independent developmental controls and value in measuring both root types in lab screens. Strong temporal and edaphic effects on mature root traits, and a lack of understanding of root trait changes during plant development, are major challenges in creating controlled-environment root screens for mature root traits in the field.

## Teaser

Root traits of young, vegetative plants grown in four controlled-environment screens were correlated with mature root traits measured from field-grown plants; correlations were few and inconsistent across trials and season.

## Introduction

### Value and expression of root traits in the field at maturity in wheat

Root traits offer a largely untapped resource for improved yield in wheat. In rainfed wheat production systems, plants are often dependent on stored soil water for some or all of their water inputs (Passioura, 1972; Lilley and Kirkegaard, 2011). Deeper mature roots that access stored soil water deep in the profile after flowering could contribute to higher yields (Manschadi *et al.*, 2006; Reynolds *et al.*, 2007) as this late season water is primarily used in filling grains, hence increasing harvest index and yield (Passioura and Angus, 2010). Phenotyping mature roots to depth in the field is possible (Wasson *et al.*, 2014; Rich *et al.*, 2016); however, it is constrained by the time wheat takes to reach maturity and the strong influence of the soil physical and chemical conditions on the mature root system phenotype (Manschadi *et al.*, 2006; Wasson *et al.*, 2012; Rich and Watt, 2013). Additionally, affordable and accurate high-throughput direct and indirect root phenotyping methods for the field are still in development (Wasson *et al.*, 2016; Slack *et al.*, 2018, Preprint; Li *et al.*, 2019). The development of high-throughput controlled-environment seedling root screens presents an opportunity to, at least partially, circumvent the current lack of field screening options (Richards *et al.*, 2010; Wasson *et al.*, 2012; Watt *et al.*, 2013).

Root systems are predisposed to a certain shape due to underlying genetics, with individual root growth patterns (tropism, branching, and elongation rates) contributing to the architecture of the complete system. As soon as roots emerge, soil physical conditions also influence root system development, with numerous biotic and abiotic factors (i.e. gravity, temperature, water, oxygen and nutrient availability, and soil biota) contributing to the final system architecture (Rich and Watt, 2013). Mature architecture will also be affected by timing and initiation of lateral and adventitious roots which are regulated by nutrient and hormonal signals (Atkinson *et al*., 2014). Mature root architecture in the field results from a combination of these internal and external signals. Controlled-environment screens for traits such as deep rooting focus on seedling and young plant vegetative stage phenotypes, because beyond that point the roots are too large for most laboratory pots and glasshouse facilities. To inform our understanding of roots, seedling root phenotypes should be correlated with root architecture in later development stages and performance in the field, in spite of all these enviromental and developmental influences.

### Relationship between young and mature and controlled-environment and field root traits

Numerous studies report relationships between growth of seedling root traits measured in controlled conditions and yield and other agronomic performance indicators in the field. Few, however, directly relate root traits from the controlled environment to root traits of mature plants in the field, particularly in relation to traits for water capture. In wheat, Watt *et al.* (2013) measured primary root traits in rolled tubes of germination paper and compared these with root system depth in the field when plants had two or five leaves on the main stem, plus at anthesis. Positive significant relationships were found between the seedling screen and roots in the field at earlier growth stages (*r*^2^=0.63 and 0.79, respectively); however, no relationship was found at anthesis. There was also no relationship between the field seedling and field mature root systems, indicating that plant age and phenology is an important contributor to relationships between controlled environment and field, not only the growing conditions. Bai *et al.* (2019) also used rolled tubes of germination paper on 637 wheat lines; root data from these were used to select 72 lines for seedling root penetration studies. Seedling root phenotypes of 72 lines were then correlated to mature root maximum depth (from core break counts of 1 m soil cores) over two seasons. They found no relationships between seedling phenotypes from either laboratory screen and their measure of root depth in the field. The ability of wheat roots to overcome mechanical resistance was also studied by Botwright Acuña *et al.* (2007). They showed that genotypes with the ability to penetrate a wax barrier in pot experiments tended to also have deep roots in the field, but found strong interactions with the field site. They concluded that the screen was inconclusive for prediction of general field performance. The emergence angle of primary axile roots in controlled-environment screens has been compared with field roots in several studies. In wheat, Oyanagi *et al.* (1993) measured the relationship between primary root angle in mesh baskets in pots with an index denoting the proportion of deep versus shallow roots in the field. They found a significant relationship between their pot screens of 7-day-old seedlings and root distribution in the field at the stem elongation stage (*r*^2^=0.78). Using a similar basket screen, a relationship between the emergence angle [measured at 36 days after sowing (DAS)] and the root system depth of 95-day-old field-grown rice has been demonstrated (Uga *et al.*, 2011). Similarly, Manschadi *et al.* (2008) found that Australian wheats that were best adapted to the northern region of Australia had a narrow root angle as seedlings, whereas wheats best adapted to the Western Australia wheat-growing region had a broad root angle. This correlated with the reliance on stored soil water in the north versus reliance on in-season winter rainfall in Western Australia.

### Approach of this study: field to lab

**Fig. 1.**
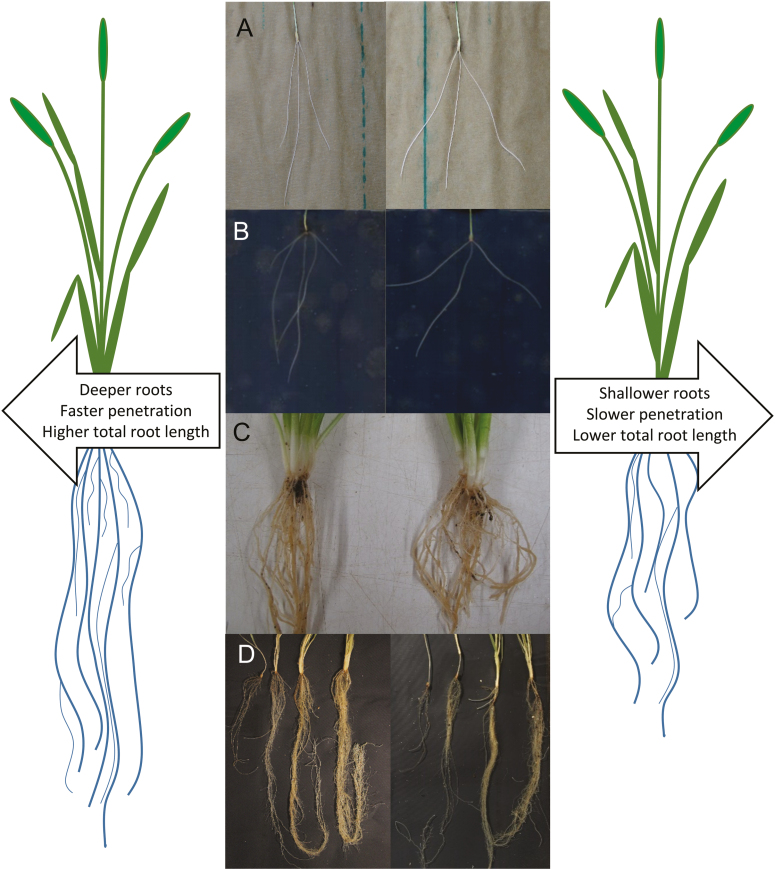
Screening for early rooting phenotypes and assessing their relationship to mature root phenotypes. Genotypes with narrow root emergence angles and high early root vigour (examples shown on the left) are hypothesized to result in narrower, deeper root systems at maturity, whereas broader angled and less vigorous seedlings (right) may result in wider shallower mature root systems. Root angle was assessed using three different methods: on primary roots sandwiched between two sheets of germination paper (A), primary roots grown between two layers of 3 mm thick agar (B), and nodal and primary root angle of roots grown in soil in buried net baskets (C). Seedling root vigour (D) was assessed in plants grown in 0.5 m×90 mm tubes harvested at 13, 20 27, and 34 DAS.

In this study, field experiments were used to establish relationships to controlled-environment screens, in a retrospective field to lab approach. Deep-rooting phenotypes measured under field conditions (Rich *et al.*, 2016) were compared with the phenotypes of young plants which were grown in four commonly used and widely available root phenotyping screens under controlled conditions (Fig. 1). The four screens used were soil tubes to measure early root vigour, harvested at four intervals (Chochois *et al.*, 2015). Root angle was measured via three different screens: germination paper (Watt *et al.*, 2013); agar (Bengough *et al.*, 2004; Manschadi *et al.*, 2008); and net baskets in soil (Oyanagi *et al.*, 1993). To ascertain if root traits measured in seedlings under controlled conditions correlated to mature root traits in the field, we focused on two main hypotheses. First, we tested whether genotypes showing early root vigour at the seedling stage would have deep root traits at maturity in the field. Secondly, we tested whether genotypes with narrower primary and nodal root emergence angles would have deeper mature root systems than those with wide root angles under field conditions.

## Materials and methods

### Root terminology

**Fig. 2.**
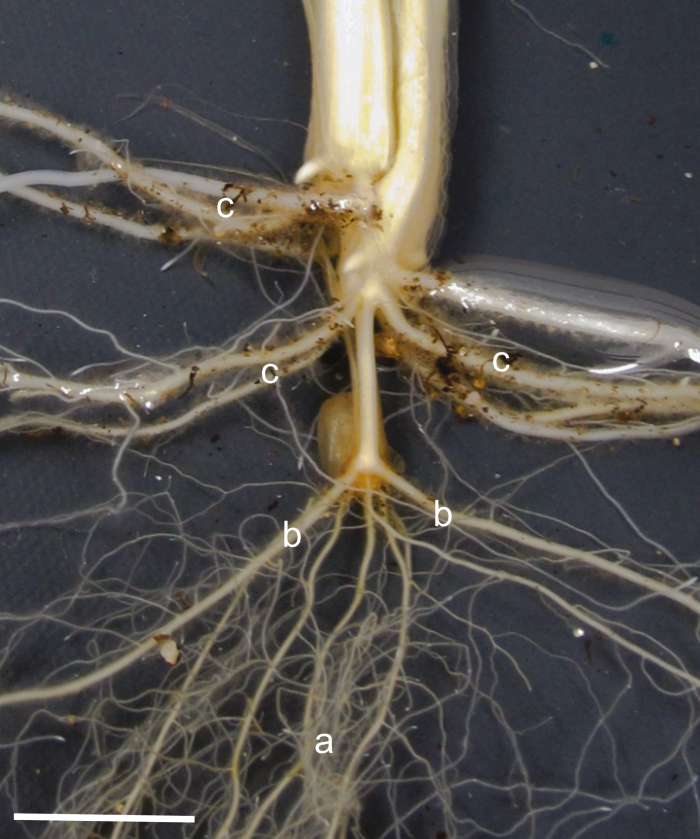
Wheat root system of a wheat plant at 35 DAS, grown under non-limiting conditions in a 0.5 m deep tube. Primary axile roots (a) develop first, followed by the coleoptile node axile roots (b) and the leaf node axile roots (c). Scutellar node axile root cannot be differentiated from fine secondary branches in this photo. Scale bar=10 mm.

Herein we follow the root classifications of Watt *et al.* (2008). Wheat has two distinct root systems (Fig. 2). The primary axile roots (PRs) emerge first from primodia within the seed, and the nodal axile roots (NRs) emerge later from leaf nodes. The NRs can be further separated into those emerging from foliar leaf nodes, termed leaf nodal roots (LNRs) or those arising from the coleoptile node just above the seed, termed coleoptile nodal roots (CNRs). Herein we are also including the single fine root that often arises from a primordium in the scutellum behind the epiblast (scutellar node axile root) with the main PR.

### Seed

Wheat genotypes used are listed in Supplementary Table S1 at *JXB* online; these are 20 genotypes selected from root coring field trials (Rich *et al.*, 2016). For all controlled-environment experiments, seed size was standardized, with only seed weighing 0.045±0.005 g being used.

### Plant growth conditions

All screens, aside from the agar-based root angle screen, were performed in the same growth cabinet (Conviron, Canada) at CSIRO Black Mountain Laboratories (Canberra, Australia), at 18±2 °C day temperatures and 15 °C night temperatures. During soil-based experiments, soil temperature was logged (Gemini Data Loggers, TinyTalk TK-0063) and had a day/night fluctuation of 16/14.5 °C. Experiments were performed under 11 h full light (1340±181 µmol m^−2^ s^−1^); lights were stepped up and down over a 0.5 h period.

### Screen 1. Soil tubes for root development and vigour through time to six leaves

This study used 0.5 m tall tubes constructed from PVC piping with a 90 mm diameter (Supplementary Fig. S1A). Tubes were filled with a soil/sand mix (Boyer *et al.*, 2010) sieved to 2 mm. Fertilizer (Aboska) was added at a concentration of 1 g l^–1^ post-sieving. Tubes were filled to a bulk density of 1.34±0.01 g cm^–3^. Two seeds were sown embryo down at a depth of 4 cm in each tube. Emergence was recorded and plants were thinned at the one-leaf stage to a single plant per tube. In 95% of tubes a seedling that emerged at 7 d was chosen. Plants were supplied with non-limiting water throughout the experiment.

The tubes were arranged in the controlled-environment cabinet in a randomized complete block design with five replicates of each line for each harvest time. The entire experiment was surrounded with another layer of tubes sown with a commercial variety. Plants were harvested at 13, 20, 27, and 34 DAS (Fig. 1D). Harvest times correspond to two, three, four, and six leaves on the main stem. In order to maintain a similar soil temperature in the adjacent tubes, at the end of each harvesting day emptied tubes were refilled with soil and replaced in the growth cabinet.

At harvest, leaf number was recorded, the tube base cap was removed, and the intact soil column was slid onto mesh trays. Soil was gently washed from the roots, and whole plants were transferred to 50% ethanol then stored at 4 °C until processed. To process, the plants were transferred to a large tray of water, then roots were carefully untangled using plastic forceps. The numbers of PR, CNRs, and LNRs were recorded, noting scutellar and coleoptile tiller roots separately (Fig. 2); only roots longer than 2 mm were counted. The length of the longest root of each type was recorded manually with a ruler and, during the 27 d (4 leaves) harvest, the axile lengths of all PRs and CNRs were recorded, plus the distance between the root base and onset of branching was measured; we scored branching by dividing the total scanned root length of the PRs and CNRs (axile and branch roots) by the total length of the root axis of that root type. The root systems of plants harvested 27 DAS were dissected into component root types and scanned at a resolution of 400 dpi on a flatbed scanner (EPSON) equipped with a transparency unit to avoid any shadowing (Regent, Quebec, Canada). Images were then processed using WinRhizo™ (Regent, Quebec, Canada) to ascertain total root length (TRL; the sum of scanned lengths of axes and branch roots). Individual root types and complete shoots were oven dried (60 °C) for 72 h to obtain dry mass.

### Screen 2. Paper pouches for primary root number and angle at one leaf

Germination paper (SDB1924, Anchor Paper Company, USA) ‘pouches’ were created by portrait orientating the papers and placing seed embryo down in the centre, 27 mm from the top. A second sheet of germination paper was laid over the top and the seed held in place using two plastic-coated paperclips. This ‘pouch’ was then fixed to a steel rod with paper clips, and 50 pouches were hung into a custom-made black Perspex box (0.48×0.26×0.28 m) filled with water so the lowest 20 mm of the pouches were submerged. Two pieces of paper (blue blotter-paper, Anchor Paper Company, USA) were attached to the inside of the box to stop the roots touching the plastic box sides (Supplementary Fig. S1B). Two empty pouches were placed at either end of the box. The top of the box was covered with plastic wrap and aluminium wrap, except for a strip 25 mm to either side of where the seeds were sown. Boxes were then kept in a dark, 4 °C room for 48 h before being transferred to the growth cabinet. We measured eight replicates of each genotype.

At harvest (7 d post-planting), the length of the single leaf was measured and intact PRs were photographed using a digital Canon 70D SLR camera. Images were analysed for root axis lengths and angles in Workspace (v3.3.0). Data produced in Workspace were checked for accuracy against manual measurement of axial length and angle of 20 images.

### Screen 3. Agar chambers for primary root number and angle at one leaf

Seed sized at the CSIRO laboratories was transferred to the Queensland Alliance for Agriculture and Food Innovation (QAAFI), where the agar-based screening was conducted.

The growth angle of the first pair of PRs and the number of PRs of wheat seedlings were measured using gel-filled root observation chambers based on methods developed from Bengough *et al.* (2004) and Manschadi *et al.* (2008). Chambers were constructed from two plates of clear glass, each measuring 210×300×3 mm. Sterilized agar (Sigma Type A; 2% w/v) was poured onto each plate and allowed to gel. Surface-sterilized wheat seed was imbibed with sterile deionized water for a few hours and then placed on wet blotting paper and kept at room temperature for 2 d to allow germination. Two germinated seeds were placed ~80 mm apart, and 50 mm from the top edge of the agar on one plate. The second plate was then carefully placed on top of the first to create a narrow air space of ~2.5 mm between the agar layers in which the seed was held by the agar layers. Plates were then taped together and vertically mounted. The seeds were oriented vertically with the radicle facing downwards (Supplementary Fig. S1C).

The agar-filled chambers were then kept in a plant growth cabinet at 15 °C for 5 d in the dark until the first leaf emerged at the top of the chambers, followed by growth under constant temperature of 15 °C with a 12/12 h dark/light regime. The light intensity in the growth cabinet at plant height was 220 µmol photons m^−2^ s^−1^. Light was excluded from the root observation chambers using black PVC plastic covers, except during observations. At 8 d after seed transfer, the PRs visible through the clear glass were scanned using a flatbed scanner (HP Scanjet 4670). The total number of PRs was recorded. The growth angle from the vertical of each root in the first pair of PRs (PR 2 and 3) to emerge after the first PR was measured within the first 3 cm of root from the seed (the basal part of the roots). Specifically designed computer software allowed rapid measurement of PR angle from the digital images as described in Manschadi *et al.* (2008).

### Screen 4. Soil baskets for primary and nodal root number and angle at the eight-leaf stage

The PVC tubes, soil, and packing methods were as described in Screen 1. Similarly, five replicate tubes were placed in a randomized complete block design, with a buffer row of tubes sown around. For this experiment, a 50 mm diameter×50 mm high net basket was buried 5 mm below the soil surface in the centre of the tube (Supplementary Fig. S1D). Seeds were pre-germinated by vernalizing at 4 °C for 4 d, then incubated at 20 °C for 1 d. A single germinated seed was then carefully sown 30 mm deep in the centre of each basket. Plants were grown with non-limiting water for 42 d. At harvest, leaf number was recorded and then the intact soil column was slid onto a mesh tray. Depth of sowing was checked using a ruler, noting any deviations from the intended distance of 25±2 mm from the open bottom of the basket; only two plants were not sown at the correct depth and they were excluded from the study. Soil was washed from the roots and basket, ensuring that roots remained emergent from the hole in the basket that they had grown through. After washing, the emergence hole of each PR and NR was recorded. Roots emerging through the bottom had an angle of 0–37° from vertical; those emerging through the subsequent rows of holes in the basket had angle ranges of 44–56, 63–79, 87–102, or 108–119° from vertical. During analysis, these were pooled to 0–37, 44–79, and >87° from vertical.

### Field experiments for root system phenotypes at grain maturity

The four screens were compared with field experimental data published in Rich *et al.* (2016) (Supplementary Table S1), obtained from independent experiments over 2 years in south-eastern Australia; different sites were used in 2012 and 2013, although they were located within 2 km of each other near Leeton, NSW. Trials were sown into a full soil water profile. Briefly, the 20 genotypes were sown in hill plots (dense tufts of ~30 seeds) in 2012 and 2013 and in 4 m^2^ plots in 2013. Post-harvest soil coring to 2 m was conducted and root traits were ascertained via the core break method. For data on variation and range of root traits, see Rich *et al.* (2016); traits used in this study are: maximum depth (the deepest 0.1 m long core segment where roots were detected); maximum depth of 90% of the roots (to remove bias of single roots that have descended deeply through a pore or crack); TLR in the core (the sum of root length as calculated from the root length density for each 0.1 m core segment); and root penetration rate (calculated as maximum depth over the growing degree days to anthesis).

### Statistical analysis

Data were collated and analysed in the R environment using the ‘reshape2’ and ‘dplyr’(Wickham, 2007; Wickham and Francois, 2014) packages. Plots and statistical analysis were also conducted in GraphPad Prism (v6.00, GraphPad Software, La Jolla CA, USA). Pearson’s correlation was used to generate matrixes of correlations between the root angle Screens 2, 3, and 4, and between all four controlled-environment screens and the field experiment phenotypes across both years. Unless otherwise stated, statistical significance was at *P*=0.05.

## Results

### Screen 1. Soil tubes (for root development and vigour through time to six leaves)

**Fig. 3.**
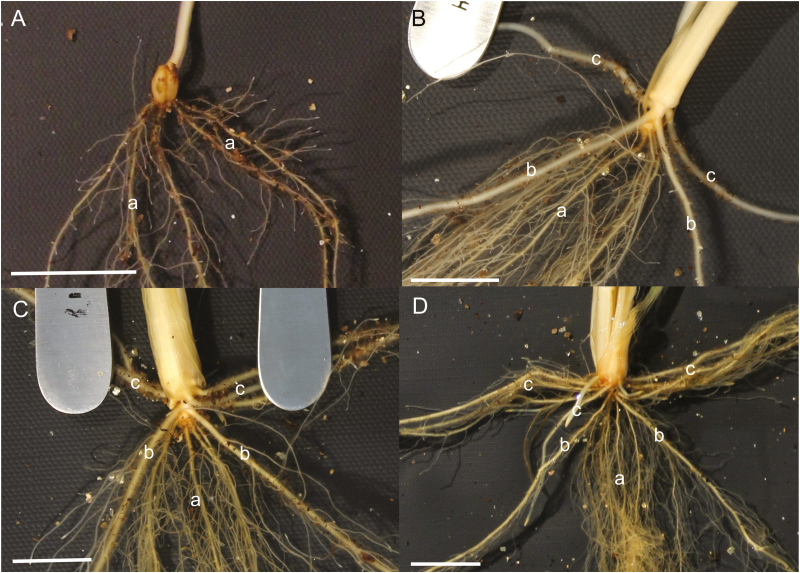
Root system of C306 at 13 (A), 20 (B), 27 (C), and 34 (D) DAS, grown under non-limiting conditions in a 0.5 m deep tubes. Primary roots (a) develop first, followed by the coleoptile nodal roots (b) and the leaf nodal roots (c). Scale bar=10 mm.

Across the four harvests at 13, 20, 27, and 34 DAS, shoot phenotypes had no significant variation among genotypes; at all harvests there was no significant difference in leaf stage between the genotypes, and variation in plant shoot dry weight was not significant at any time point (data not shown). Significant variation was, however, found among root phenotypes in root number, length, emergence, development of branch roots, and angle of emergence. Root development progressed in a similar manner across genotypes, illustrated using photos of Indian line C306 in Fig. 3. The timing of root development varied slightly between genotypes; and generally the PRs had emerged at 13 DAS (Fig. 3A) and CNRs developed between 13 and 20 DAS, during which all genotypes also started to develop LNRs (only HI 1500 and NIL3-14 had developed LNRs in all replicates; number of LNRs <5; Fig. 3B). The period between 20 and 34 DAS showed continued elongation of all root types and further initiation of LNRs (Figs 3, 5), with some genotypes having up to 25 LNRs by 34 DAS [genotypes with a mean higher than 20 LNRs were Gregory, COW(W)-1 and HS420]. Most plants produced five PRs; however, this did vary from four to seven (Table 1; Supplementary Table S2). Approximately 30% of seeds across all genotypes produced a scutellar node axile root. Genotypes predominantly grew either one or two CNRs, although the range was 0–3 CNRs (Table 1; Supplementary Table S2).

**Fig. 4.**
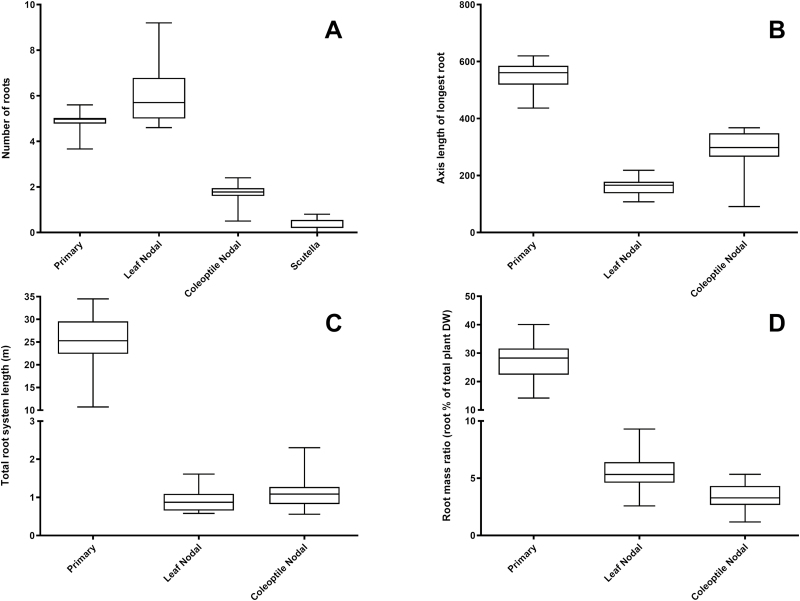
Variation in root traits for 20 wheat varieties at 27 DAS; number of roots (A), axis length of longest root (B), total root system length (C), and root mass ratio. Number of roots (A) and axis length of the longest root (B) were determined manually at time of harvest. Total root system length (C) was determined by scanning root systems and analysing with WinRhizo software. Root mass ratio (D) was determined by the dry mass of root to shoot tissue. Data from 18 wheat genotypes grown under non-limiting conditions in a 0.5 m deep tubes (*n*=5/genotype).

**Fig. 5.**
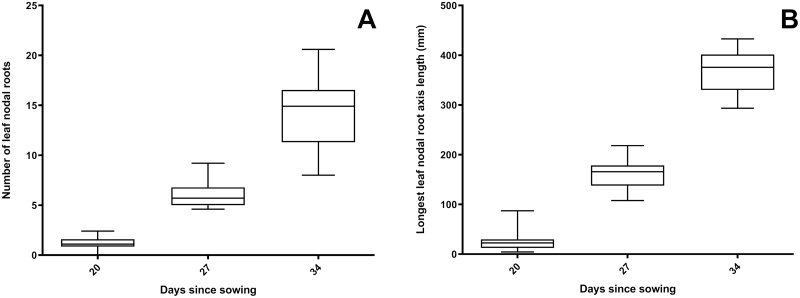
Variation in initiation and elongation of leaf nodal roots for 20 wheat varieties between 20 and 34 DAS into 0.5 m deep tubes. Number of leaf nodal roots (A) and axis length of longest nodal root (B) were determined manually at time of harvest.

**Table 1.** Genotypic variation in wheat root traits at 27 DAS

		Minimum	Maximum
Total root system	TRL	12.49±1.65	36.81±3.61
	(m per plant)		
	Root mass ratio	18.79±1.97	50.36±0.86
	(% of shoot DW)		
Primary roots	Number (omit scutella)	4	7
	Maximum axis length	436.75±48.20	619.80±20.30
	(mm)		
	TRL	10.72±1.40	34.51±2.93
	(m per plant)		
	Root mass ratio	14.17±0.47	40.04±2.27
	(% of shoot DW)		
	PR system elongation	18.51±0.83	29.17±0.56
	13 DAS–27 DAS (mm d^−1^)		
	Branching score	8.46±1.59	15.86±1.56
	(total length/axis length)		
Coleoptile nodal roots	Number	0	3
	maximum axis length	199.40±36.00	367.60±23.40
	(mm)		
	TRL	0.68±0.84	2.30±0.28
	(m per plant)		
	Root mass ratio	1.18±0.16	5.34±0.71
	(% of shoot DW)		
	CNR system elongation	20.24±3.28	36.71±2.07
	20 DAS–34 DAS (mm d^−1^)		
	Branching score	1.32±0.15	3.85±0.35
	(total length/axis length)		
Leaf nodal roots	Number	3	12
	Maximum axis length	107.75±8.29	195.50±16.90
	(mm)		
	TRL	0.58±0.10	1.61±0.71
	(m per plant)		
	Root mass ratio	2.58±0.81	7.20±0.72
	(% of shoot DW)		
	LNR system elongation	16.31±4.9	30.90±3.16
	27 DAS–34 DAS (mm d^−1^)		

Data presented are the minimum and maximum mean (*n*=5) from 20 wheat genotypes grown under non-limiting conditions in soil in 0.5 m deep tubes. Plants were gently shaken from the tubes and washed free of soil by hand. Total root length (TRL) is the sum of root lengths of all axes and branch roots from WinRhizo analyses; axis length is the length of the main axis, measured manually with a ruler.

The 20 genotypes studied showed large variation in the detailed root phenotypes measured at 27 DAS (Fig. 4; Table 1; Supplementary Table S2). The root system was a significant contributor to biomass, as compared with the shoot. The root system mass was as much as 50% of the shoot biomass at 27 DAS, although in some genotypes this was as low as 20%, indicating substantial variation in allocation patterns. The main contributor to root biomass was the PR (Table 1); LNRs and CNRs were smaller contributors to biomass, weighing only 5% and 7%, respectively, of shoot biomass (Table 1). Both length and number of roots contributed to the variation in root biomass.

There was a 3-fold variation in TRL, which, as with biomass, was predominantly driven by the extensive branching of the PRs. Interestingly, there was no correlation between the number of PRs (ranging from 4 to 7) and the total scanned length; the PR axis length (driven by the root elongation rate) had a slight positive correlation with TRL; however, this was also not significant. Root elongation rates of the individual root types was highly variable (Table 1), with elongation rates ranging >2-fold from 16.31±4.9 mm d^−1^ to 36.71±2.07 mm d^−1^. Despite the large variation, the different root types had similar elongation rates.

First-order and second-order branch roots were the primary drivers of TRL. We scored branching in PRs and CNRs at 27 DAS by dividing scanned root length by the total axile length of each root type. In PRs, each centimetre of axile root had up to 15.86±1.6 cm (Westonia) of branch root length, while the CNRs had a far lower branch length of 1.32±0.15–3.85±0.35 cm per centimetre of main axile (Table 1). In PRs we also measured the length down the axile from the root base to the start of secondary branching. No significant difference in this length at 27 DAS was found between the genotypes; overall mean length from the PR root base to the start of branching across all genotypes was 277.3±6.3 mm.

### Screen 2. Paper pouches (for primary root number and angle at one leaf)

**Fig. 6.**
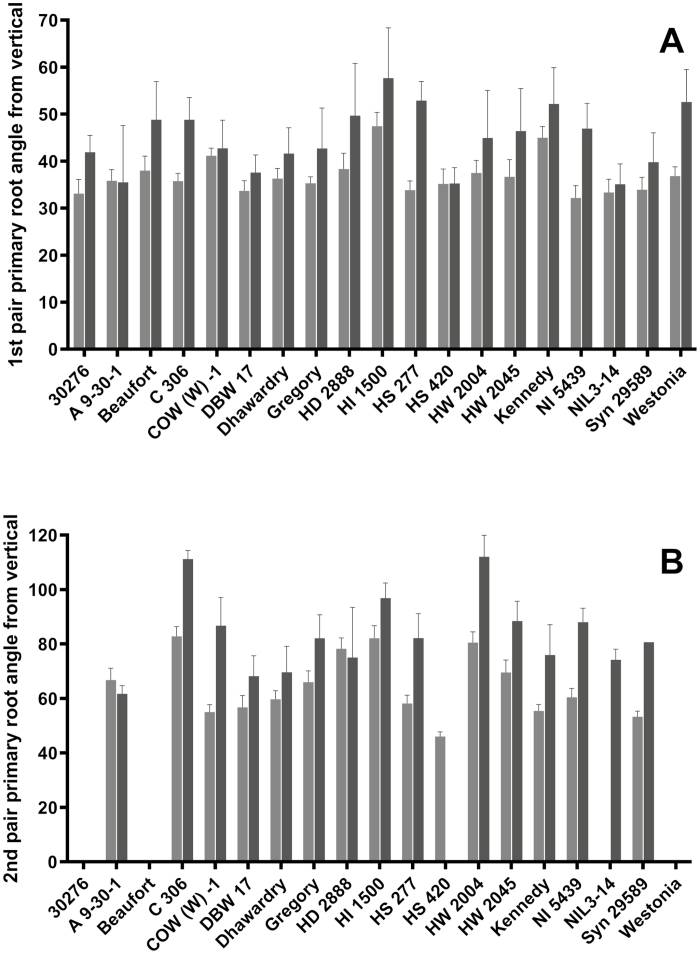
Primary root (PR) angle from vertical of the first root pair (A) and the second root pair (B) measured in 3 mm thick chambers glass filled with agar (grey) and on germination paper (black). Twenty wheat genotypes were grown; however, in four genotypes, most replicates did not produce second root pairs so were not included (30276, Beaufort, HS 420, Beaufort, and Westonia). Genotypes are ordered alphabetically. Bars are shown with SE; *n*=5/genotype.

A total of 19 genotypes were analysed (Janz was removed due to poor germination). The angle of the first pair of PRs (PR 2 and 3) from the vertical ranged from 35° to 57.7±10.71°, being the lowest in A9-30-1, HS 420, and NILS-14 and highest in HI1500 (Fig. 6A); however, there is no significant difference between genotypes. Only 15 genotypes had three replicates or more with a second pair of PRs (PR 4 and 5). The angle between the second pair of PRs varied significantly among genotypes. Angles ranged from 61.7±2.9° in A9-30-1 to >110° (111.22°±3.1° in C306 and 112.04°±8.9 in HW 2004; Fig. 6B). Pairwise comparisons showed that the second pair of PRs in HW 2004 and C306 were significantly wider (~45°) than those of Dhawardry, DBW 17, and A9-30-1. There was no relationship between the angle of the first and second PR pair. No correlation was found between leaf or root length and any PR angles.

### Screen 3. Agar chambers (for primary root number and angle at one leaf)

In contrast to Screen 2 in paper pouches, the angle of the first pair of PRs varied significantly among genotypes in the agar chambers (*P*<0.05). The angle from vertical ranged from 32.17±2.60° in NI 5439 to 47.42±2.96° in HI 1500 (Fig. 6A). Multiple pairwise comparisons substantiated that genotype HI 1500 expressed a significantly wider angle between PR 2 and PR 3 (47.42±2.96°) than the narrow-angled genotypes (30276, DBW 17, HS 277, NI5439, NIL3-14, and Syn 25859), which had narrower angles by ~14° (Fig. 6A). Similar to the paper pouch (Screen 2), some genotypes did not produce >3 replicates with PR 4 and 5, namely 03627, Beaufort, Westonia, HS 277, and Syn 25859. Among the 15 genotypes with a second PR pair (PR 4 and 5) for analysis, the angle from the vertical ranged from 46.0±1.76° in HS 420 to 82° in both C306 and HI 1500 (Fig. 6B), and variation between genotypes was highly significant (*P*<0.0001). As in the paper pouch screen, first and second PR pairs did not have angles that correlated significantly. Generally, angles were slightly narrower in the agar chambers compared with the paper pouches.

### Screen 4. Soil baskets (for primary and nodal root number and angle at the eight-leaf stage)

**Fig. 7.**
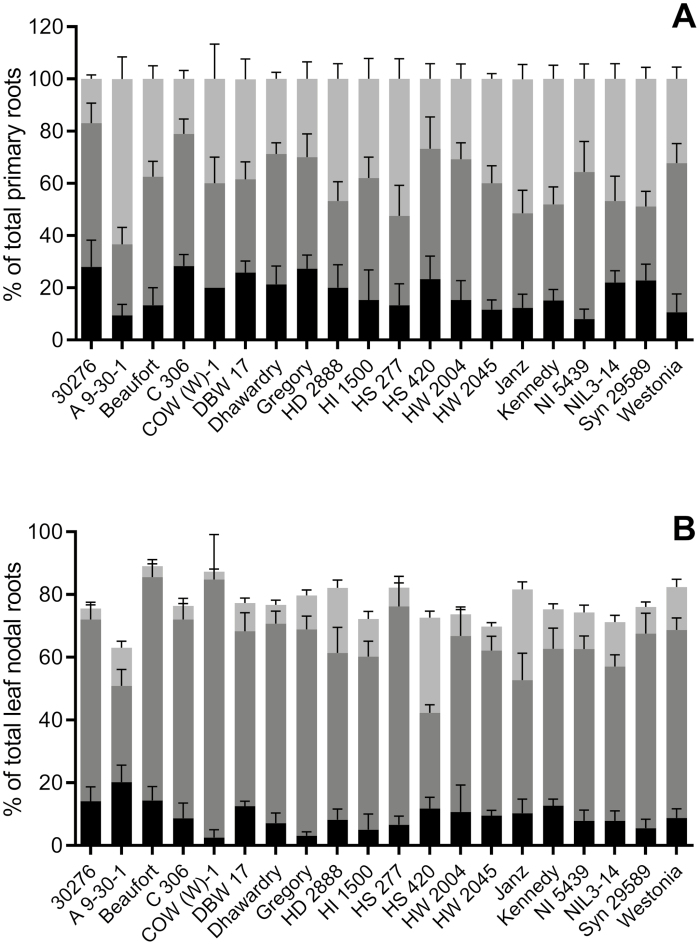
3D root angle from the vertical of the primary (A) and nodal (B) wheat roots grown in soil through a net basket. Graphs show the percentage of total roots emerging at narrow angles (black 0–37°), medium angles (dark grey 44–79°), and wide angles (pale grey >87°) from the vertical. Remaining nodal roots (to bring the total to 100%) are those nodal roots not yet long enough to emerge through the basket. Genotypes are ordered alphabetically. Bars are shown with SE; *n*=5/genotype.

The plants in the soil basket screen were assessed at 42 DAS; 5 weeks older than the plants in the paper pouch and agar chambers, and therefore the NR angle as well as the PR angle could be assessed. The soil, 3D basket configuration, and advanced plant age meant that the CNRs and LNRs were crowded and difficult to differentiate. Therefore, roots were classified only as PRs or NRs, and expressed as a percentage of the total number for each angle range (Fig. 7; Supplementary Table S3). Plants produced 4–7 PRs, with most producing five. Nodal root production was more varied, with plants growing between seven and 24 NRs (mean=12.70±0.68).

Roots emerging through the base of the net basket had an angle of 0–37° from vertical, which was our narrowest class. C306, 30276, Gregory, and DBW 17 had >25% of their total PRs in this narrow class (Fig. 7A). Most genotypes, including those with some narrow PRs, had a significant number of PRs emerging at wider root angles; Janz, HS 277, and A9-301 all had >60% of their PRs growing at angles wider than 87°. Nodal roots in all genotypes tended to grow out at mid-range angles. Only five genotypes did not have at least 50% of nodal roots growing in the 44–79° range (Fig. 7). Only A9-30-1 had >20% of its NRs growing at less than 37°, and only Janz and HS420 produced >25% of total NRs growing at angles wider than 87°. There was no significant relationship between PR and NR angles, within or between genotypes.

### Correlations between angles measured in Screens 2, 3, and 4

The paper pouch and agar chamber screens were positively and significantly correlated for PR angles for the first pair (*P*=0.0095) and the second pair (*P*=0.018). Wider PR angles in the soil baskets were significantly positively correlated with the angle of the second pair of PRs measured in the paper pouches (44–79° in soil, *P*=0.0058; and >87° in soil, *P*=0.012). This was not the case for PR angles measured in the agar chambers. Nodal root angles in the soil baskets showed no significant relationship with PR angles measured on the young plants of the paper pouch and agar chamber screens.

### Correlations between controlled-environment Screens 1–4 and field root phenotypes

We examined the correlation between root traits ascertained from our controlled-environment screens: Screen 1 soil tubes (root development through time to 34 DAS), Screen 2 and 3 paper pouches and agar chambers (PR angles at 8 DAS), and Screen 4 soil baskets (PR and NR angles and numbers at 42 DAS); and the field phenotypes from cores of the same genotypes measured at maturity over 2 years (Supplementary Table S1; maximum root system depth, maximum depth of 90% of roots, TRL in the core, and root penetration rate). There was significant year to year and site to site variation in results for root traits between the field trials (Rich *et al.*, 2016). We did find significant relationships between some phenotypes from the controlled-environment screen and phenotypes from field screens; however, no seedling trait showed a significant relationship to a mature rooting trait across the three field trials (Table 2; Supplementary Tables S4 and S5 for full correlation matrices with *r*^2^ and *P*-values).

**Table 2.** Significant correlation *R*^2^ values between field root trait correlations and the traits derived from developmental seedling screen and from root angle screens in paper rolls or in agar

Field derived traits (from Rich *et al.*, 2016)	2012 Maximum depth	2013 Maximum depth	2013 Maximum depth	2012 90th percentile maximum depth	2013 90th percentile maximum depth	2013 90th percentile maximum depth	2012 Root penetration rate	2013 Root penetration rate	2013 Root penetration rate	2012 Total root length in core	2013 Total root length in core	2013 Total root length in core
Plot type	Hill	Hill	4 m^2^	Hill	Hill	4 m^2^	Hill	Hill	4 m^2^	Hill	Hill	4 m^2^
**Seedling root traits**												
***Root development in soil***												
PR longest axis length 13 DAS		–0.57**					–0.53*	–0.53*			–0.45*	
PR longest axis length 20 DAS										0.48*		
LNR longest axis length 20 DAS			0.61**			0.45*			0.57**			
LNR longest axis length 27 DAS			0.58**			0.47*			0.52*			
CNR longest axis length 27 DAS	0.50*		0.47*		0.60**							
PR/shoot DW ratio												-0.67*
Number of LNRs 20 DAS			0.61**			0.45*			0.57**			
PR branching score				–0.55*								
CNR elongation 20–34 DAS											0.49*	
***Paper root angle screen***												
Second PR pair angle				0.62**			0.48*					
***Soil root angle screen***												
% NR <37°			–0.73***			–0.62**			-0.72***			
% NR 44–79°			0.53*									
% PR <37°	0.55*			0.45*	0.54*					0.44*		
% PR >87°				–0.69***	–0.49*						–0.58**	

For field traits, genotypes were sown in either hill plots (Hill; dense tufts of ~30 seeds) or 4 m^2^ plots (4 m^2^). More detail on the field trials has been published by Rich *et al.* (2016). A full correlation matrix and table of *P*-values is included in Supplementary Tables S1 and S2. Abbreviations: days after sowing (DAS); primary roots (PRs); nodal roots (NRs); leaf nodal roots (LNRs); coleoptile nodal roots (CNRs). ***P<0.001, **P<0.01, *P<0.05.

From the traits measured in soil tubes, fast root elongation at the seedling stage (as represented by the longest axile length of each root type) showed the strongest relationships to deep rooting traits at maturity. PR, CNR, and LNR axile lengths at 20 and 27 DAS significantly correlated to mature root traits in some trials (Table 2). Early development of LNRs, as indicated by a high number of LNRs at 20 DAS, also seems to give some indication of potential depth in the field, although total numbers of LNRs developed (counts at 27 and 34 DAS) showed no relationships (Supplementary Tables S4, S5). At 13 DAS, axile lengths of PRs had negative relationships with mature depth traits, suggesting that very early PR vigour does not necessarily predict genotypes with greater root system depth at maturity.

The root emergence angles as measured in the paper pouch and agar chamber screens showed very little correlation to mature deep rooting traits; only the angle of the second root pair from the vertical on paper was correlated to rooting depth and penetration, and only in the 2012 field trials. The root emergence angles measured in the soil baskets showed stronger relationships to mature field traits. Genotypes, such as C306 and Gregory, with a high percentage of narrow angled PRs (<37° from the vertical; Supplementary Table S2) expressed some deep rooting traits in some field trials (Table 2). Narrow-angled NRs showed a negative relationship to deep rooting mature traits, as did very wide-angled PRs (>87° from the vertical; Table 2).

## Discussion

This study targeted seedling root system traits that would potentially correlate to deep root characteristics in mature field-grown plants. We found relationships between some seedling root traits and field root traits in particular seasons and sites (Table 2); however, extensive root trait variation in the field from site to site and year to year somewhat confounded our ability to obtain clear and consistent correlations with seedling and mature root traits. This lack of strong relationships between seedling and mature root traits highlights a key impediment to the development of an effective seedling proxy for mature root traits; the strong influence of soil physical, chemical, and edaphic factors on root traits (Rich and Watt, 2013) results in site and seasonal variation strongly impacting mature root phenes.

The field trial sites in the 2 years were located in close proximity and on similar soils; however, weather patterns in the 2 years were very different. The 2013 season received twice as much in-season rainfall as in 2012, which as well as a low total rainfall received a third of the in-season rainfall a week before harvest when the crop had essentially already senesced. Both 2013 trials show extensive root proliferation at mid-range depths (~0.5 m), and the maximum depth achieved in these trials tended to be less than in 2012. It is likely that there was little value in proliferation in the mid-range depths in 2012 as the water profile was retreating from the surface throughout the season (Rich *et al.*, 2016).

### Root angle screens were strongly correlated with each other; however, the soil root angle screen showed stronger relationships to mature field traits than the germination paper and agar screens

Three of the tested root screens measured root angle. We trialled two screens which allowed us to measure the PR angle as grown on a 2D plane (Screen 2, paper pouches and screen 3, agar chambers); these screens have been commonly used due to their speed and ease of execution. We also trialled a method of screening both PRs and NRs growing in soil in the 3D plane (Oyanagi *et al.*, 1993). Root angle in cereals has been of particular interest within the literature of late, in part due to the prevalence of quantitative trait loci (QTLs) being found for aspects of this trait (Omori and Mano, 2007; Uga *et al.*, 2011; Mace *et al.*, 2012; Christopher *et al.*, 2013; Lopez *et al.*, 2017). While there is a general belief that a narrower root system relates to a deeper mature root system in the field, there is limited published evidence to support this. In maize and rice, there is some evidence linking seedling root angle to root traits in the field; however, in wheat, this link has not been definitively established. Manschadi *et al.* (2008) established indirect evidence of seedling narrow root angles and deep rooting through demonstrating that certain wheat genotypes with a narrow PR angle tended to develop more roots directly under the plant 33 DAS. Oyanagi *et al.* (1993) show a significant relationship between seedling root angle screens and root distribution in the field; however, this was at the stem elongation stage, and it has been demonstrated by Watt *et al.* (2013) that strong relationships between seedling root screens and field measures early in development can disappear by anthesis.

Our finding that genotypes having a higher percentage of wide-angled PRs (>87°) produced negative correlations to some deep rooting traits in mature plants in the field gives some support to the hypothesis that roots emerging at narrow angles lead to deeper root architecture at maturity. However, narrow-angled NRs also had a negative correlation with deep rooting at maturity. In both cases, these relationships were only found in some trials with some deep-rooting traits (Table 2), again highlighting the strong edaphic effect. From the three angle screens we recorded no trait that produced a consistent proxy for traits measured during field coring at maturity. However, of all recorded angle traits, the candidates most likely to correlate with field measurements were from the 3D soil screen. Interestingly, in our experiments, the angle of the first PR pair, which is the most commonly used, showed no significant correlations to the field (Table 2). Of the three controlled-environment root angle screening methods, the soil-based screen grows the roots in the most natural conditions, in soil and not forcing roots into the 2D plane. However, it is more labour intensive to set up and uses far more space in a controlled-environment cabinet than the other two methods so would probably need adapting (possibly along the lines of Richard *et al.*, 2015) to become truly useful as a screen for large numbers of genotypes.

### Longest root axis length was more likely to produce a relationship with any mature traits than scanned total root length

We ran the developmental growth screen (Screen 1; soil tubes) in order to capture a picture of the timing of root type development and to allow us to screen the same trait at various points in seedling development to explore if correlations to mature roots changed. The most time-consuming aspects of this screen were separation of root types and scanning for total root system length. However, these measures showed no correlations with any of the field traits measured (Table 2). The closest correlations with data for field traits were found for the axis length of all three root types. As hypothesized, in most cases, having greater early vigour (long axis length) at the seedling stage had positive correlations with deeper rooting traits at maturity. However, the opposite was true for the length of the initial PR at 13 d after sowing which showed a negative relationship to mature deep-rooting traits. The axis length of NRs showed relationships to the field from 3 weeks after sowing. Early development of LNRs also gave some indication of potential mature depth in the field (Table 2).

NRs were a significant contributor to both root biomass and depth (Table 1); however, many screens tend to overlook these roots, focusing on the PRs. NR axial numbers are far higher than those of PRs; in this study at 34 days, at a total of 4–7 PRs had emerged, yet up to 25 LNRs were already present and, by maturity in the field, an individual can have as many as 50 LNRs (personal observation). Although NRs emerge later than PRs, at the 34 d harvest in our controlled-experiments some NRs had already elongated to similar lengths as the PRs (Fig. 5) and therefore by maturity could be a significant contributor to the deep-rooting system of the plant. How much elongation rates of the roots types differ under different conditions is not fully understood and nor is it clear if either main root axis is important at depth or if it is branch roots which are more significant. The stronger relationships we found with seedling nodal root phenes and mature field traits suggest that these root types may play an important role in deep root architecture; however, distinguishing a PR from an NR at depth under field conditions is extremely difficult (Watt *et al.*, 2008).

### No single seedling trait could be identified as an effective screen; however, a combined trait screen could capture useful genetic variation

With no single seedling trait showing strong correlations to mature deep rooting traits across trials, a screen that captures several seedling phenes could be an option. The candidates highlighted here could be used in the design of a combined trait screen. These experiments were not designed for multiple regression analysis; however, simple ranking gives an indication of the usefulness of collecting several traits. Very few genotypes perform consistently well across traits; if we examine the ranks of genotypes that were deep rooted at maturity in Rich *et al.* (2016), they rank in the top 20% for many seedling traits but are mid-range or even low ranked for other traits. Looking across several traits, however, deep-rooted genotypes do tend to have a larger number of highly ranked seedling screening results than other genotypes that were found to be more shallowly rooted in the field.

Our results highlight a key issue in developing an effective seedling proxy for mature root traits: the strong influence of soil physical, chemical, and edaphic factors on root traits (Rich and Watt, 2013). While we found relationships between various screening traits and root traits in the field at maturity, rarely did these correlations occur across field trials. It seems doubtful that a single, simple, affordable, and strongly correlated screen can be developed using any of the tested methods, if indeed at all.

Although disappointing, this finding is not unexpected for the reasons outlined above. To provide additional context, it is worthwhile considering what above-ground traits in wheat are related to crop performance that breeders may use to select for grain yield. Apart from poor early establishment, breeders do not generally select for above-ground seedling traits as these are not correlated with yield. Time of flowering and plant height are generally the first above-ground traits selected as these are related to adaptation and yield. It is useful to consider the comprehensive study reported by Quail *et al.* (1989) who measured >50 traits on plants in an early generation (F_3_) in the glasshouse. These plants were multiplied to F_7_ and F_8_, and were then grown in large plots in replicated yield traits in favourable environments over several years. Plant height and harvest index were the only traits significantly correlated with yield in all trials.

### Conclusion: root traits at the vegetative stage show low correlation with genetic variation in mature wheat root traits in the field

We have correlated numerous root traits from four screens of seedling and early vegetative wheat roots, measuring growth and angle phenotypes, with four mature root traits measured from field-grown plants. We found some correlations between seedling root traits and mature field-grown roots; however, these correlations were inconsistent across trials and season.

The variation seen in the field trials demonstrates the strong effect of temporal and edaphic factors on mature root traits; this, combined with our current lack of understanding of root trait changes as the plant develops through tillering, stem elongation, anthesis, and grain fill, is the major challenge to be overcome in the development of a seedling proxy for mature root traits. Given the results reported here, the most effective way to screen for mature root depth may be the use of high-throughput, field-based techniques. Methods in development include high-throughput soil coring methods (Wasson *et al*., 2014, 2016), mature root crown analysis methods (Slack *et al.*, 2018, Preprint), and non-destructive, above-ground indicators of crop ‘health’ after flowering such as cooler canopy temperature and/or high normalized difference vegetation index (Li *et al.*, 2019). These have been shown to be effective to identify higher yielding wheat genotypes with deeper roots, but further work remains to ascertain the most efficient and effective phenotyping method.

## Supplementary data

Supplementary data are available at *JXB* online.

Fig. S1. Methods used in the seedling screens.

Table S1. List of genotypes used in field and controlled-environment studies.

Table S2. Genotypic variation in root traits of 18 wheat genotypes 27 d after sowing.

Table S3. Genotypic variation in root emergence angle of 20 diverse wheat genotypes 42 d after sowing.

Table S4. Correlation *P*-values of the developmental seedling screen and root angle screens to field root trait correlations.

Table 5. Correlation *r*^2^ values of the developmental seedling screen and root angle screens to field root trait correlations.

eraa201_suppl_Supplementary_Figure_S1_Tables_S1-S5

## Abbreviations

CNR: coleoptile nodal root
DAS: days after sowing
LNR: leaf nodal root
NR: nodal root
PR: primary axile root
TRL: total root length

## References

[CIT0001] Atkinson JA, Rasmussen A, Traini R, Voß U, Sturrock C, Mooney SJ, Wells DM, Bennett MJ 2014 Branching out in roots: uncovering form, function, and regulation. Plant Physiology 166, 538–550. 25136060 10.1104/pp.114.245423 PMC4213086

[CIT0002] Bai C, Ge Y, Ashton RW, et al 2019 The relationships between seedling root screens, root growth in the field and grain yield for wheat. Plant and Soil 440, 311–326.

[CIT0003] Bengough AG, Gordon DC, Al-Menaie H, Ellis RP, Allan D, Keith R, Thomas WTB, Forster BP 2004 Gel observation chamber for rapid screening of root traits in cereal seedlings. Plant and Soil 262, 63–70.

[CIT0004] Botwright Acuña TL, Pasuquin E, Wade LJ 2007 Genotypic differences in root penetration ability of wheat through thin wax layers in contrasting water regimes and in the field. Plant and Soil 301, 135–149.

[CIT0005] Boyer JS, Silk WK, Watt M 2010 Path of water for root growth. Functional Plant Biology 37.

[CIT0006] Chochois V, Vogel JP, Rebetzke GJ, Watt M 2015 Variation in adult plant phenotypes and partitioning among seed and stem-borne roots across *Brachypodium distachyon* accessions to exploit in breeding cereals for well-watered and drought environments. Plant Physiology 168, 953–967. 25975834 10.1104/pp.15.00095 PMC4741322

[CIT0007] Christopher J, Christopher M, Jennings R, Jones S, Fletcher S, Borrell A, Manschadi AM, Jordan D, Mace E, Hammer G 2013 QTL for root angle and number in a population developed from bread wheats (*Triticum aestivum*) with contrasting adaptation to water-limited environments. Theoretical and Applied Genetics 126, 1563–1574. 23525632 10.1007/s00122-013-2074-0

[CIT0008] Li X, Ingvordsen CH, Weiss M, Rebetzke GJ, Condon AG, James RA, Richards RA 2019 Deeper roots associated with cooler canopies, higher normalized difference vegetation index, and greater yield in three wheat populations grown on stored soil water. Journal of Experimental Botany 70, 4963–4974. 10.1093/jxb/erz232 PMC6760272 31089708

[CIT0009] Lilley JM, Kirkegaard JA 2011 Benefits of increased soil exploration by wheat roots. Field Crops Research 122, 118–130.

[CIT0010] Lopez JR, Erickson JE, Munoz P, Saballos A, Felderhoff TJ, Vermerris W 2017 QTLs associated with crown root angle, stomatal conductance, and maturity in Sorghum. Plant Genome 10, 1–12. 10.3835/plantgenome2016.04.0038 28724080

[CIT0011] Mace ES, Singh V, Van Oosterom EJ, Hammer GL, Hunt CH, Jordan DR 2012 QTL for nodal root angle in sorghum (*Sorghum bicolor* L. Moench) co-locate with QTL for traits associated with drought adaptation. Theoretical and Applied Genetics 124, 97–109. 21938475 10.1007/s00122-011-1690-9

[CIT0012] Manschadi A, Hammer G, Christopher J, deVoil P 2008 Genotypic variation in seedling root architectural traits and implications for drought adaptation in wheat (*Triticum aestivum* L.). Plant and Soil 303, 115–129.

[CIT0013] Manschadi AM, Christopher J, deVoil P, Hammer GL 2006 The role of root architectural traits in adaptation of wheat to water-limited environments. Functional Plant Biology 33, 823–837. 32689293 10.1071/FP06055

[CIT0014] Omori F, Mano Y 2007 QTL mapping of root angle in F2 populations from maize ‘B73’×teosinte ‘*Zea luxurians*’. Plant Root 1, 57–65.

[CIT0015] Oyanagi A, Nakamoto T, Wada M 1993 Relationship between root growth angle of seedlings and vertical distribution of roots in the field in wheat cultivars. Japanese Journal of Crop Science 62, 565–570.

[CIT0016] Passioura J 1972 The effect of root geometry on the yield of wheat growing on stored water. Australian Journal of Agricultural Research 23, 745–752.

[CIT0017] Passioura JB, Angus JF 2010 Improving productivity of crops in water-limited environments. Advances in Agronomy 106, 37–75.

[CIT0018] Quail K, Fischer R, Wood J 1989 Early generation selection in wheat. I. Yield potential. Australian Journal of Agricultural Research 40, 1117–1133.

[CIT0019] Reynolds M, Dreccer F, Trethowan R 2007 Drought-adaptive traits derived from wheat wild relatives and landraces. Journal of Experimental Botany 58, 177–186. 17185737 10.1093/jxb/erl250

[CIT0020] Rich SM, Wasson AP, Richards RA, et al 2016 Wheats developed for high yield on stored soil moisture have deep vigorous root systems. Functional Plant Biology 43, 173–188. 32480451 10.1071/FP15182

[CIT0021] Rich SM, Watt M 2013 Soil conditions and cereal root system architecture: review and considerations for linking Darwin and Weaver. Journal of Experimental Botany 64, 1193–1208. 23505309 10.1093/jxb/ert043

[CIT0022] Richard CA, Hickey LT, Fletcher S, Jennings R, Chenu K, Christopher JT 2015 High-throughput phenotyping of seminal root traits in wheat. Plant Methods 11, 13. 25750658 10.1186/s13007-015-0055-9 PMC4351910

[CIT0023] Richards RA, Rebetzke GJ, Watt M, Condon AGT, Spielmeyer W, Dolferus R 2010 Breeding for improved water productivity in temperate cereals: phenotyping, quantitative trait loci, markers and the selection environment. Functional Plant Biology 37, 85–97.

[CIT0024] Slack S, York LM, Roghazai Y, Lynch J, Bennett M, Foulkes J 2018 Wheat shovelomics II: Revealing relationships between root crown traits and crop growth. BioRxiv 10.1101/280917 [Preprint].

[CIT0025] Uga Y, Okuno K, Yano M 2011 *Dro1*, a major QTL involved in deep rooting of rice under upland field conditions. Journal of Experimental Botany 62, 2485–2494. 21212298 10.1093/jxb/erq429

[CIT0026] Wasson A, Bischof L, Zwart A, Watt M 2016 A portable fluorescence spectroscopy imaging system for automated root phenotyping in soil cores in the field. Journal of Experimental Botany 67, 1033–1043. 26826219 10.1093/jxb/erv570 PMC4753854

[CIT0027] Wasson AP, Rebetzke GJ, Kirkegaard JA, Christopher J, Richards RA, Watt M 2014 Soil coring at multiple field environments can directly quantify variation in deep root traits to select wheat genotypes for breeding. Journal of Experimental Botany 65, 6231–6249. 24963000 10.1093/jxb/eru250 PMC4223987

[CIT0028] Wasson AP, Richards RA, Chatrath R, Misra SC, Prasad SV, Rebetzke GJ, Kirkegaard JA, Christopher J, Watt M 2012 Traits and selection strategies to improve root systems and water uptake in water-limited wheat crops. Journal of Experimental Botany 63, 3485–3498. 22553286 10.1093/jxb/ers111

[CIT0029] Watt M, Magee LJ, McCully ME 2008 Types, structure and potential for axial water flow in the deepest roots of field-grown cereals. New Phytologist 178, 135–146. 18221246 10.1111/j.1469-8137.2007.02358.x

[CIT0030] Watt M, Moosavi S, Cunningham SC, Kirkegaard JA, Rebetzke GJ, Richards RA 2013 A rapid, controlled-environment seedling root screen for wheat correlates well with rooting depths at vegetative, but not reproductive, stages at two field sites. Annals of Botany 112, 447–455. 23821620 10.1093/aob/mct122 PMC3698392

[CIT0031] Wickham H 2007 Reshaping data with the reshape package. Journal of Statistical Software 21, 1–20.

[CIT0032] Wickham H, Francois R 2014 dplyr: a grammar of data manipulation, R package version 0.1 http://CRAN.R-project.org/package=dplyr.

