# Supplementary material for: Root phenotypes of young wheat plants grown in controlled environments show inconsistent correlation with mature root traits in the field
Source: J Exp Bot. 2020 Apr 29;71(16):4751–62. doi: 10.1093/jxb/eraa201 (PMC7410186; doi:10.1093/jxb/eraa201)
Supplement: eraa201_suppl_Supplementary_Figure_S1_Tables_S1-S5 [file eraa201_suppl_supplementary_figure_s1_tables_s1-s5.pdf]

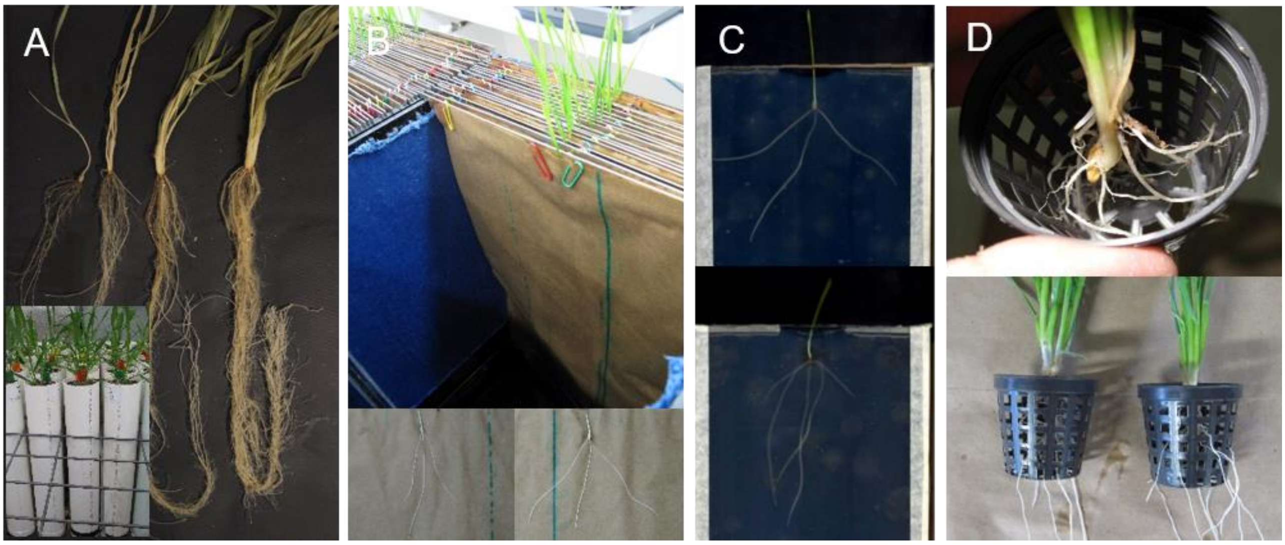

Supplementary Figure 1: Methods used in the seedling screens. Developmental growth screen (A), plants were grown in 0.5 m x 90 mm pots (insert) and harvested at 13, 20, 27 and 34 days after sowing, at harvest the intact soil column was slid onto mesh trays and soil was gently washed from the roots. Root angle was assessed using three different methods. Plants were grown for 7 days sandwiched between two sheets of germination paper (B) so initial seminal root angles could be measured (insert shows roots at harvest). Seminal root angle was also measured on roots grown between two layers of 3 mm thick agar (C). Nodal and seminal root angle was assessed on roots grown in soil by determining root emergence through the holes in buried net baskets (D).

Supplementary Table 1: List of genotypes used in field and controlled environment studies. Rooting depth data is presented from field experiments in Rich *et al.* (2016). Root depth data is from post-harvest soil coring to 2 m with root depth ascertained via the core break method, maximum depth (the deepest 0.1 m long core segment where roots were detected) and maximum depth of 90 % of the roots.

Name	Group	2012 Hill plots						2013 4 m^2 plots						2013 Hill plots					
		Maximum depth			Maximum depth 90 % roots			Maximum depth			Maximum depth 90 % roots			Maximum depth			Maximum depth 90 % roots		
30276	Breeding line	140.0	±	5.0	113.8	±	6.5	112.0	±	3.7	76.0	±	6.8	105.0	±	5.7	93.3	±	3.3
A 9-30-1	Indian cultivar	137.5	±	8.4	127.5	±	18.9	98.0	±	6.0	65.0	±	6.5	105.0	±	8.7	75.0	±	6.5
Beaufort	Australian commercial CV	136.3	±	8.7	110.0	±	6.5	118.0	±	10.3	76.0	±	5.1	103.8	±	7.1	60.0	±	NA
C 306	Indian cultivar	140.0	±	5.7	111.4	±	8.6	118.0	±	4.9	88.0	±	3.7	111.3	±	7.4	78.3	±	6.0
COW (W) - 1	Indian cultivar	143.8	±	6.8	113.3	±	9.2	136.0	±	6.8	84.0	±	7.5	110.0	±	5.0	83.3	±	6.7
DBW 17	Indian cultivar	141.4	±	5.1	105.0	±	5.6	110.0	±	8.1	77.5	±	8.5	110.0	±	6.5	76.7	±	8.0
Dhawardry	Indian cultivar	138.8	±	6.9	100.0	±	3.8	128.0	±	4.9	85.0	±	6.5	107.5	±	7.5	80.0	±	10.0
Gregory	Australian commercial CV	146.3	±	6.3	116.0	±	9.8	124.0	±	5.8	85.0	±	6.5	108.8	±	6.7	85.0	±	10.4
HD 2888	Indian cultivar	136.3	±	9.6	101.4	±	9.1	126.0	±	6.0	93.3	±	8.8	101.3	±	8.3	80.0	±	6.3
HI 1500	Indian cultivar	138.8	±	11.1	108.8	±	7.4	144.0	±	9.7	94.0	±	7.5	117.5	±	8.0	85.0	±	5.6
HS 277	Indian cultivar	133.8	±	8.9	100.0	±	7.6	118.0	±	6.6	85.0	±	6.5	97.5	±	4.5	90.0	±	NA
HS 420	Indian cultivar	134.3	±	5.7	111.7	±	7.0	112.0	±	8.6	80.0	±	11.5	114.3	±	6.1	90.0	±	4.1
HW 2004	Indian cultivar	141.3	±	6.4	107.1	±	5.2	120.0	±	9.3	80.0	±	8.4	114.3	±	5.7	82.5	±	9.5
HW 2045	Indian cultivar	128.6	±	11.4	108.6	±	8.3	102.0	±	7.7	72.0	±	7.3	101.3	±	5.2	70.0	±	7.1
Janz	Australian commercial CV	131.3	±	4.8	100.0	±	5.2	104.0	±	3.7	76.0	±	6.0	110.0	±	4.2	82.5	±	7.5
Kennedy	Australian commercial CV	126.3	±	7.3	98.3	±	4.0	100.0	±	5.5	72.0	±	5.8	113.8	±	4.6	87.1	±	3.6
NI 5439	Indian cultivar	140.0	±	7.1	96.0	±	12.1	124.0	±	5.8	85.0	±	5.0	112.5	±	7.3	88.0	±	4.9
NIL3-14	Breeding line	142.5	±	8.6	101.7	±	6.0	126.0	±	12.9	86.7	±	8.8	105.0	±	5.0	70.0	±	3.2
Syn29589	Breeding line	135.7	±	7.8	97.5	±	4.8	120.0	±	7.5	86.7	±	3.3	111.3	±	4.0	85.0	±	2.9
Westonia	Australian commercial CV	127.5	±	5.3	107.5	±	7.0	110.0	±	6.3	80.0	±	4.5	101.3	±	5.8	80.0	±	10.0

Supplementary Table 2: Genotypic variation in root traits of 18 wheat genotypes 27 days after sowing. Plants were grown under non-limiting conditions in soil in 0.5 m deep tubes. Plants were gently shaken from the tubes and roots washed free of soil by hand. Total root length (TRL) is the sum of root lengths of all axes and branch roots from WinRhizo analyses; axis length is the length of the main axis, measured manually with a ruler. Due to some samples being misplaced during processing, data on all phenes is not available for genotypes HI1500 and HW2004. Data presented are means and SE, n = 5 unless * where n = 4;

Name	Total Root System		Primary seminal roots (PR)						
	Total root length (m/plant)	Root mass ratio (% of total shoot DW)	Number of PR	Scutella present (% of 5 reps)	Maximum PR axis length (mm)	Total PR length (m/plant)	PR system elongation 13DAS-27DAS (mm d-1)	PSR branching score (cm total length/ axis cm)	Root mass ratio (% of total shoot DW)
30276	22.42 ± 2.06	36.48 ± 1.09	5 ± 0	25	522.5 ± 6.74	21.16 ± 2.04	24.15 ± 0.85	9.47 ± 0.50	29.51 ± 1.08
A 9-30-1	12.49 ± 1.66	18.79 ± 0.88	5.2 ± 0.2	60	501 ± 19.03	10.72 ± 1.40	24.92857 ± 1.09	na	14.17 ± 0.47
Beaufort	24.89 ± 2.09	37.84 ± 1.13	4.75 ± 0.25	0	551.75 ± 29.51	22.81 ± 2.09	24.3 ± 1.90	10.55 ± 2.41	26.92 ± 1.07
C306	27.63 ± 2.45	*32.10 ± 1.24	5 ± 0	0	586.2 ± 23.62	25.00 ± 2.45	18.51429 ± 0.83	8.52 ± 0.87	*22.41 ± 0.92
COW (W) -1	28.28 ± 5.68	32.19 ± 2.89	4.8 ± 0.2	40	577.2 ± 57.73	26.51 ± 5.39	26.58571 ± 0.70	10.94 ± 2.37	24.91 ± 0.89
DBW 17	27.15 ± 2.14	40.66 ± 0.83	5 ± 0	20	552.4 ± 22.84	25.29 ± 1.75	25 ± 0.14	10.87 ± 0.43	32.90 ± 0.83
Dhawardry	16.27 ± 1.88	36.00 ± 2.66	4.8 ± 0.2	80	500.4 ± 25.47	14.69 ± 1.69	22.85714 ± 1.41	na	25.51 ± 2.72
Gregory	29.93 ± 2.90	32.95 ± 1.26	5.2 ± 0.2	20	544.2 ± 18.57	26.34 ± 2.47	22.05714 ± 0.44	12.82 ± 1.21	21.97 ± 0.71
HD 2888	26.41 ± 3.37	*36.79 ± 0.30	5 ± 0	0	619.8 ± 20.31	24.83 ± 3.58	27.42857 ± 1.99	8.46 ± 1.60	*22.29 ± 6.58
HI 1500	na	na	5 ± 0	40	583.6 ± 14.33	na	19.21071 ± 2.74	na	na
HS 277	33.50 ± 3.46	*50.36 ± 0.61	5 ± 0	20	517.6 ± 25.86	31.71 ± 2.65	22.15714 ± 0.10	14.20 ± 0.50	*22.09 ± 2.75
HS 420	26.86 ± 2.16	*26.75 ± 0.50	4.8 ± 0.4	40	576.8 ± 27.44	25.31 ± 2.08	25.24286 ± 1.00	10.90 ± 0.84	*40.04 ± 2.27
HW 2004	na	na	5 ± 0	20	544.6 ± 17.95	na	23.72857 ± 2.90	na	na
HW 2045	32.32 ± 1.77	40.67 ± 0.91	5 ± 0	0	581 ± 17.25	29.18 ± 1.46	24.01429 ± 1.27	11.12 ± 0.43	29.71 ± 1.80
Janz	21.28 ± 1.55	21.47 ± 2.21	5 ± 0	25	436.75 ± 48.19	20.04 ± 1.57	29.17143 ± 0.56	12.90 ± 0.57	32.18 ± 3.54
Kennedy	25.58 ± 3.22	*37.57 ± 3.73	4.4 ± 0.4	80	586.4 ± 15.87	24.36 ± 3.50	24.58571 ± 1.18	11.29 ± 0.01	31.47 ± 4.55
NI 5439	31.80 ± 0.86	35.28 ± 1.07	4.8 ± 0.2	20	598.6 ± 15.01	30.76 ± 1.07	27.46429 ± 0.62	13.72 ± 0.48	*29.07 ± 0.95
NIL3-14	34.45 ± 1.79	42.43 ± 1.18	5 ± 0	20	586.2 ± 34.33	32.19 ± 1.39	24.12857 ± 1.20	11.22 ± 0.67	33.98 ± 1.66
Syn29589	28.73 ± 2.17	37.04 ± 1.64	5 ± 0	60	569.6 ± 32.32	27.22 ± 1.55	25.5 ± 1.18	na	29.44 ± 1.70
Westonia	38.37 ± 3.61	35.23 ± 1.22	5 ± 0	80	506.4 ± 119.15	34.51 ± 2.94	23.8 ± 2.39	15.86 ± 1.56	27.47 ± 1.47

Name	Leaf Nodal Roots (LNR)				
	Number of LNR	Maximum LNR axis length (mm)	Total LNR length (m/plant)	LNR system elongation 27DAS-34DAS (mm d-1)	Root mass ratio (% of total shoot DW)
30276	4.75 ± 0.75	107.75 ± 8.29	0.59 ± 0.06	24.87 ± 2.82	4.02 ± 0.12
A 9-30-1	5.60 ± 1.12	140.60 ± 31.40	0.68 ± 0.22	21.29 ± 6.18	2.58 ± 0.81
Beaufort	6.75 ± 0.63	195.50 ± 16.88	1.03 ± 0.06	20.96 ± 1.79	7.20 ± 0.72
C306	5.00 ± 0.71	165.00 ± 8.21	0.94 ± 0.02	24.31 ± 1.60	*4.76 ± 0.20
COW (W) -1	6.80 ± 0.97	167.80 ± 35.94	1.08 ± 0.24	24.83 ± 1.29	4.76 ± 0.89
DBW 17	4.60 ± 0.24	123.20 ± 6.63	0.67 ± 0.14	28.38 ± 1.77	4.13 ± 0.36
Dhawardry	4.80 ± 0.20	192.40 ± 22.84	0.72 ± 0.11	19.46 ± 11.13	6.22 ± 1.30
Gregory	7.80 ± 0.58	180.40 ± 14.11	1.28 ± 0.30	18.96 ± 3.93	5.80 ± 0.73
HD 2888	5.20 ± 0.37	166.40 ± 36.79	0.62 ± 0.02	27.96 ± 2.00	*5.46 ± 0.63
HI 1500	5.80 ± 0.37	218.20 ± 12.52	na	28.26 ± 2.33	na
HS 277	5.20 ± 0.58	136.80 ± 12.82	0.58 ± 0.10	27.87 ± 5.15	*4.84 ± 0.32
HS 420	7.40 ± 0.75	195.40 ± 17.38	1.00 ± 0.08	28.88 ± 1.75	*5.81 ± 1.30
HW 2004	6.00 ± 0.71	143.40 ± 18.33	na	26.56 ± 0.55	na
HW 2045	6.50 ± 0.65	173.00 ± 7.38	1.23 ± 0.18	24.48 ± 4.71	5.62 ± 0.44
Janz	5.00 ± 0.00	163.75 ± 26.18	0.87 ± 0.21	30.90 ± 3.16	9.29 ± 3.08
Kennedy	5.00 ± 0.45	116.60 ± 13.33	0.61 ± 0.01	16.31 ± 4.93	4.06 ± 0.03
NI 5439	5.60 ± 0.24	157.60 ± 18.55	0.82 ± 0.08	21.87 ± 0.47	*5.20 ± 0.26
NIL3-14	6.80 ± 0.58	172.60 ± 15.32	1.15 ± 0.05	27.53 ± 1.06	7.16 ± 0.39
Syn29589	6.60 ± 0.98	168.80 ± 21.13	0.87 ± 0.24	26.67 ± 1.93	5.00 ± 0.91
Westonia	9.20 ± 1.24	136.40 ± 37.65	1.61 ± 0.34	21.82 ± 2.54	6.95 ± 0.94

Name	Coleoptile Nodal Roots (CNR)					
	Number of CNR	Maximum CNR axis length (mm)	Total CNR length (m/plant)	CNR system elongation 27DAS-34DAS (mm d-1)	CNR branching score (cm total length/ axis cm)	Root mass ratio (% of total shoot DW)
30276	1.75 ± 0.25	228.00 ± 4.02	0.67 ± 0.06	33.52 ± 3.34	2.42 ± 0.09	2.94 ± 0.18
A 9-30-1	2.00 ± 0.00	273.60 ± 20.57	1.09 ± 0.15	21.55 ± 1.23	na	2.79 ± 0.33
Beaufort	1.75 ± 0.25	275.50 ± 28.64	1.05 ± 0.27	20.24 ± 3.28	1.77 ± 0.07	3.72 ± 1.04
C306	1.60 ± 0.24	351.00 ± 28.47	1.69 ± 0.02	27.99 ± 1.13	2.64 ± 0.10	*4.93 ± 0.12
COW (W) -1	2.20 ± 0.37	293.60 ± 35.91	1.46 ± 0.39	28.07 ± 0.81	2.75 ± 0.70	3.83 ± 0.20
DBW 17	1.60 ± 0.24	312.80 ± 38.64	1.20 ± 0.39	33.61 ± 0.48	2.35 ± 0.39	3.62 ± 0.85
Dhawardry	1.80 ± 0.20	293.80 ± 30.14	0.87 ± 0.18	22.66 ± 1.39	na	4.26 ± 1.06
Gregory	2.40 ± 0.24	361.00 ± 13.31	2.30 ± 0.29	28.56 ± 0.04	3.81 ± 0.42	5.18 ± 0.23
HD 2888	1.20 ± 0.20	367.60 ± 23.37	0.96 ± 0.22	25.76 ± 2.73	2.11 ± 0.11	*2.78 ± 0.01
HI 1500	1.80 ± 0.20	347.40 ± 33.94	na	28.10 ± 0.75	na	na
HS 277	2.00 ± 0.00	302.60 ± 39.40	1.22 ± 0.70	22.00 ± 4.87	2.54 ± 0.53	*2.71 ± 0.55
HS 420	1.60 ± 0.24	289.40 ± 56.38	1.21 ± 0.22	27.99 ± 0.31	3.25 ± 0.23	*4.51 ± 1.57
HW 2004	1.60 ± 0.24	337.40 ± 33.30	na	27.25 ± 3.31	na	na
HW 2045	2.00 ± 0.41	366.75 ± 19.53	1.92 ± 0.25	25.14 ± 6.30	3.86 ± 0.36	5.34 ± 0.71
Janz	0.50 ± 0.29	91.00 ± 71.28	0.56 ± 0.43	36.71 ± 2.07	2.64 ± 0.68	2.04 ± 1.66
Kennedy	1.80 ± 0.20	238.60 ± 18.75	0.61 ± 0.29	27.49 ± 0.72	1.79 ± 0.10	2.04 ± 0.78
NI 5439	0.50 ± 0.26	340.50 ± 35.10	0.89 ± 0.11	30.31 ± 0.42	na	*2.50 ± 0.33
NIL3-14	1.40 ± 0.24	348.80 ± 18.48	1.08 ± 0.20	28.90 ± 1.72	2.32 ± 0.21	2.90 ± 0.38
Syn29589	1.80 ± 0.20	263.20 ± 28.78	1.14 ± 0.20	30.06 ± 0.02	na	3.78 ± 0.30
Westonia	1.80 ± 0.20	199.40 ± 36.05	0.69 ± 0.08	31.62 ± 0.48	1.32 ± 0.15	1.18 ± 0.16

Supplementary Table 3: Genotypic variation in root emergence angle of 20 diverse wheat genotypes 42 days after sowing. Root angle from vertical of the primary and nodal wheat roots is presented as percent of total roots of that type emerging at narrow angles (0 °- 37 °), medium angles (44 °-79 °) and wide angles (>87°) from vertical. Root angle was assessed on 6 w old plants grown in soil under controlled conditions in 0.5 m deep tubes, germinated seed was sown at a fixed depth into a buried net plastic pot. At harvest plants were gently shaken from the tubes and washed free of soil by hand and the emergence hole in the net basket of each seminal and nodal root was recorded, allowing for root angle to be calculated. Nodal roots not yet long enough to emerge through the basket were not counted. Data is mean of 5 replicates with standard error.

Genotype	Primary roots			Nodal roots		
	0 °- 37 ° (%)	44 °- 79 ° (%)	> 87 ° (%)	0 °- 37 ° (%)	44 °- 79 ° (%)	> 87 ° (%)
30276	28.0 ± 10.2	55.0 ± 7.8	17.0 ± 1.5	14.1 ± 4.6	57.9 ± 5.5	3.5 ± 1.2
A 9-30-1	9.4 ± 4.3	27.2 ± 6.5	63.3 ± 8.5	20.2 ± 5.4	30.7 ± 5.2	12.1 ± 2.1
Beaufort	13.3 ± 6.7	49.2 ± 5.9	37.5 ± 5.1	14.4 ± 4.4	71.1 ± 5.6	3.5 ± 0.8
C 306	28.3 ± 4.4	50.6 ± 5.7	21.1 ± 3.2	8.7 ± 4.8	63.3 ± 6.8	4.4 ± 0.7
COW (W)-1	20.0 ± 0.0	40.0 ± 10.0	40.0 ± 13.3	2.5 ± 2.5	82.3 ± 7.3	2.5 ± 0.8
DBW 17	25.8 ± 4.4	35.8 ± 6.7	38.3 ± 7.8	12.5 ± 1.6	55.8 ± 5.9	9.0 ± 1.6
Dhawardry	21.3 ± 7.0	50.0 ± 4.3	28.7 ± 2.5	7.1 ± 3.3	63.6 ± 4.0	6.0 ± 1.5
Gregory	27.3 ± 5.2	42.7 ± 8.9	30.0 ± 6.5	3.1 ± 1.3	65.8 ± 4.2	10.8 ± 1.8
HD 2888	20.0 ± 8.9	33.3 ± 7.4	46.7 ± 5.8	8.2 ± 3.4	53.2 ± 8.1	20.7 ± 2.5
HI 1500	15.3 ± 11.6	46.7 ± 8.0	38.0 ± 7.8	5.0 ± 5.0	55.2 ± 4.9	12.0 ± 2.4
HS 277	13.3 ± 8.2	34.2 ± 11.7	52.5 ± 7.7	6.6 ± 2.8	69.6 ± 9.6	6.0 ± 1.5
HS 420	23.3 ± 8.8	50.0 ± 12.1	26.7 ± 5.8	11.8 ± 3.6	30.5 ± 2.6	30.3 ± 2.1
HW 2004	15.3 ± 7.4	54.0 ± 6.2	30.7 ± 5.7	10.7 ± 8.6	56.1 ± 9.2	6.9 ± 1.5
HW 2045	11.7 ± 3.7	48.3 ± 6.7	40.0 ± 2.0	9.5 ± 1.7	52.6 ± 4.6	7.7 ± 1.2
Janz	12.3 ± 5.2	36.3 ± 8.8	51.3 ± 5.6	10.3 ± 4.5	42.4 ± 8.6	28.9 ± 2.4
Kennedy	15.2 ± 4.2	36.7 ± 6.7	48.1 ± 5.2	12.7 ± 2.1	50.0 ± 6.6	12.6 ± 1.7
NI 5439	8.1 ± 3.7	56.3 ± 11.7	35.6 ± 5.7	7.9 ± 3.4	54.7 ± 4.2	11.7 ± 2.3
NIL3-14	22.0 ± 4.5	31.3 ± 9.5	46.7 ± 5.8	7.9 ± 3.1	49.1 ± 3.8	14.2 ± 2.2
Syn 29589	22.8 ± 6.2	28.3 ± 5.8	48.9 ± 4.4	5.5 ± 2.9	62.0 ± 6.5	8.5 ± 1.6
Westonia	10.7 ± 6.9	57.0 ± 7.5	32.3 ± 4.6	8.8 ± 2.9	59.9 ± 3.8	13.7 ± 2.5

Supplementary Table 4: Correlation p-values of developmental seedling screen and root angle screens to field root trait correlations. Genotypes were sown in either hill plots (dense tufts of ~30 seeds) or 4 m^2 plots. Detail of the field trials has been published in elsewhere (Rich *et al.*, 2016). Abbreviations; days after sowing (DAS); primary seminal roots (PSR); leaf nodal roots (LNR); coleoptile nodal roots (CNR). p < .001, "*** ", p < .01, "** ", p < .05, "* "

Field derived traits (from Rich et al., 2016)	2012 Maximum depth	2013 Maximum depth	2013 Maximum depth	2012 90th percentile maximum depth	2013 90th percentile maximum depth	2013 90th percentile maximum depth	2012 Root penetration rate	2013 Root penetration rate	2013 Root penetration rate	2012 Total root length in core	2013 Total root length in core	2013 Total root length in core
Plot type	hill	hill	$4m^2$	hill	hill	$4m^2$	hill	hill	$4m^2$	hill	hill	$4m^2$
**Seedling root traits**												
Root development in soil												
Total root length 27DAS	0.5491	0.2738	0.5819	0.4918	0.8405	0.5112	0.4466	0.2836	0.4043	0.7552	0.751	0.7748
CNR length 27DAS	0.0678	0.9291	0.3734	0.4389	0.0881	0.8731	0.9151	0.9549	0.5237	0.6798	0.5164	0.857
LNR length 27DAS	0.844	0.6923	0.6798	0.8776	0.8182	0.5877	0.6489	0.4852	0.4804	0.53	0.7636	0.9546
PR length 27DAS	0.6284	0.4972	0.3051	0.6677	0.9787	0.273	0.7815	0.4804	0.1181	0.5336	0.5695	0.6967
PR longest axis length 13DAS	0.9031	0.0083**	0.5949	0.2048	0.9397	0.6418	0.0161*	0.0210*	0.5952	0.2333	0.0445*	0.136
Leaf length 20DAS	0.5083	0.1789	0.6304	0.4273	0.8895	0.72	0.3391	0.0614	0.7564	0.5853	0.1946	0.5679
PR longest axis length 20DAS	0.803	0.7589	0.2847	0.6285	0.7223	0.4599	0.5944	0.7627	0.2035	0.0338*	0.8613	0.8516
number of LNR 20DAS	0.2635	0.3116	0.0040**	0.2043	0.6523	0.0480*	0.2188	0.8191	0.0080**	0.8704	0.5762	0.5399
LNR longest axis length 20DAS	0.4169	0.5959	0.3002	0.8852	0.0873	0.2484	0.5999	0.8049	0.272	0.2367	0.2158	0.6219
CNR longest axis length 20DAS	0.6066	0.5742	0.1706	0.6492	0.7443	0.356	0.5761	0.6497	0.1998	0.6668	0.243	0.1961
number of LNR 27DAS	0.7079	0.637	0.7143	0.6563	0.5342	0.6418	0.5229	0.4091	0.5722	0.5662	0.9317	0.668
LNR longest axis length 27DAS	0.3773	0.269	0.0078**	0.1641	0.8521	0.0369*	0.3996	0.7346	0.0185*	0.5107	0.7994	0.2583
CNR longest axis length 27DAS	0.0256*	0.9631	0.0363*	0.1358	0.0055**	0.4042	0.6242	0.4161	0.0896	0.3647	0.8291	0.7109
number of LNR 34DAS	0.119	0.2229	0.4536	0.9159	0.7994	0.3701	0.2381	0.9636	0.398	0.9293	0.995	0.4837
LNR longest axis length 34DAS	0.977	0.5072	0.388	0.3234	0.6606	0.1892	0.7524	0.9891	0.2088	0.3822	0.9731	0.7942
CNR/S DW ratio	0.0689	0.4568	0.3416	0.0611	0.061	0.9819	0.4036	0.4553	0.7267	0.5216	0.8918	0.7961
NR/S DW ratio	0.4565	0.7425	0.5524	0.755	0.2195	0.5855	0.5545	0.1133	0.4224	0.3844	0.3545	0.4947
PR/S DW ratio	0.3493	0.0692	0.6816	0.7294	0.6279	0.8378	0.0791	0.2017	0.9188	0.8502	0.0677	0.0176*
PR branching score	0.2241	0.5186	0.6274	0.0342*	0.0746	0.7491	0.5639	0.2751	0.8445	0.6702	0.5409	0.7155
CNR branching score	0.278	0.5462	0.8579	0.5641	0.3488	0.6796	0.4117	0.5022	0.9712	0.5011	0.9156	0.6288
PR elongation 13-27 DAS	0.955	0.743	0.4938	0.8323	0.8372	0.3533	0.6918	0.9654	0.3418	0.3923	0.739	0.197
CNR elongation 20-34 DAS	0.7152	0.0927	0.9052	0.8507	0.4597	0.536	0.156	0.2475	0.6611	0.3636	0.0283*	0.0802
LNR elongation 27-34 DAS	0.8928	0.7968	0.35	0.2239	0.7197	0.1976	0.5007	0.2533	0.2087	0.7975	0.9041	0.1367

Supplementary Table 5: Correlation r^2 values of developmental seedling screen and root angle screens to field root trait correlations. Genotypes were sown in either hill plots (dense tufts of ~30 seeds) or 4 m^2 plots. Detail of the field trials has been published in detail elsewhere (Rich *et al.*, 2016). Abbreviations; days after sowing (DAS); primary seminal roots (PSR); nodal roots (NR); leaf nodal roots (LNR); coleoptile nodal roots (CNR). p < .001, "*** ", p < .01, "** ", p < .05, "* "

Field derived traits (from Rich et al., 2016)	2012 Maximum depth	2013 Maximum depth	2013 Maximum depth	2012 90th percentile maximum depth	2013 90th percentile maximum depth	2013 90th percentile maximum depth	2012 Root penetration rate	2013 Root penetration rate	2013 Root penetration rate	2012 Total root length in core	2013 Total root length in core	2013 Total root length in core
Plot type	hill	hill	$4m^2$	hill	hill	$4m^2$	hill	hill	$4m^2$	hill	hill	$4m^2$
**Seedling root traits**												
Root development in soil												
Total root length 27DAS	-0.14	-0.26	0.13	-0.16	-0.05	0.16	-0.18	-0.26	0.2	0.07	0.08	-0.09
CR length 27DAS	0.44	-0.02	0.22	0.19	0.41	0.04	0.03	-0.01	0.16	0.1	-0.16	0.06
NR length 27DAS	-0.05	-0.1	0.1	-0.04	-0.06	0.14	-0.12	-0.18	0.18	-0.16	-0.08	0.02
PR length 27DAS	-0.12	-0.17	0.26	-0.11	-0.01	0.27	-0.07	-0.18	0.38	0.16	0.14	-0.13
PR longest axis length 13DAS	0.03	-0.57**	-0.13	-0.3	0.02	-0.11	-0.53*	-0.53*	-0.13	0.28	-0.45*	0.46
Leaf length 20DAS	0.16	-0.31	0.11	-0.19	0.03	-0.09	-0.23	-0.44	0.07	0.13	-0.3	-0.18
PR longest axis length 20DAS	-0.06	0.07	0.25	-0.12	0.08	0.18	0.13	0.07	0.3	0.48*	0.04	0.06
number of NR 20DAS	0.26	0.24	0.61**	0.3	0.11	0.45*	0.29	0.06	0.57**	0.04	0.13	0.2
NR longest axis length 20DAS	-0.21	0.14	-0.27	-0.04	-0.43	-0.3	0.14	-0.07	-0.28	-0.3	-0.32	0.18
CR longest axis length 20DAS	0.12	0.13	0.32	0.11	0.08	0.22	0.13	0.11	0.3	0.1	-0.27	0.4
number of NR 27DAS	-0.09	-0.11	0.09	-0.11	-0.15	0.11	-0.15	-0.2	0.13	-0.14	-0.02	0.14
NR longest axis length 27DAS	0.21	0.26	0.58**	0.32	0.04	0.47*	0.2	0.08	0.52*	-0.16	-0.06	0.35
CR longest axis length 27DAS	0.50*	0.01	0.47*	0.35	0.60**	0.2	0.12	0.2	0.39	0.21	0.05	-0.12
number of NR 34DAS	0.36	0.29	0.18	0.03	0.06	0.21	0.28	0.01	0.2	0.02	0	-0.22
NR longest axis length 34DAS	0.01	0.16	0.2	0.23	0.1	0.31	0.08	0	0.29	0.21	0.01	0.08
CR/S DW ratio	0.44	0.19	0.24	0.45	0.45	-0.01	0.21	0.19	0.09	0.16	0.03	0.08
NR/S DW ratio	-0.19	-0.08	0.15	0.08	-0.3	0.14	-0.15	-0.4	0.2	-0.22	-0.23	0.22
PR/S DW ratio	-0.23	0.44	-0.1	0.09	-0.12	-0.05	0.42	0.33	-0.03	0.05	0.44	-0.67*

Field derived traits (from Rich et al., 2016)	2012 Maximum depth	2013 Maximum depth	2013 Maximum depth	2012 90th percentile maximum depth	2013 90th percentile maximum depth	2013 90th percentile maximum depth	2012 Root penetration rate	2013 Root penetration rate	2013 Root penetration rate	2012 Total root length in core	2013 Total root length in core	2013 Total root length in core
Plot type	hill	hill	$4m^2$	hill	hill	$4m^2$	hill	hill	$4m^2$	hill	hill	$4m^2$
PR branching score	-0.33	-0.18	-0.14	-0.55*	-0.47	-0.09	-0.16	-0.3	-0.06	-0.12	-0.17	0.13
CR branching score	0.31	0.18	0.05	0.17	0.27	-0.12	0.24	0.2	-0.01	-0.2	-0.03	-0.19
PR elongation 13-27 DAS	0.01	0.08	-0.16	-0.05	-0.05	-0.22	0.09	-0.01	-0.22	-0.2	0.08	-0.4
CR elongation 20-34 DAS	0.09	0.39	-0.03	0.04	0.18	0.15	0.33	0.28	0.1	0.21	0.49*	-0.52
NR elongation 27-34 DAS	-0.03	-0.06	0.22	0.28	-0.09	0.3	-0.16	-0.28	0.29	0.06	-0.03	0.46
Paper root angle screen												
1st PS pair angle	-0.39	-0.07	0.22	-0.11	-0.32	0.24	0.02	-0.06	0.28	-0.15	-0.2	0.36
2nd PS pair angle	0.17	0.43	0.27	0.62**	0.35	0.33	0.48*	0.41	0.3	0.19	0.29	0.03
Agar root angle screen												
1st PS pair angle	-0.33	0.38	0.13	-0.23	-0.41	0.28	0.37	0.29	0.25	-0.31	-0.13	0.18
Soil root angle screen												
% NR <37°	-0.28	-0.12	-0.73***	-0.06	-0.16	-0.62**	-0.19	-0.01	-0.72***	-0.22	-0.2	-0.39
% NR <56°	-0.35	0.15	-0.3	-0.18	-0.31	-0.21	0.08	0.24	-0.23	-0.36	-0.09	-0.08
% NR 44-56°	0.33	-0.26	0.47*	0.16	0.16	0.4	-0.18	-0.33	0.37	0.22	0.09	0.70*
% NR 63-79°	0.06	0.02	0.29	0.16	0.1	0.05	0.12	-0.09	0.23	0.36	0.11	-0.02
% NR 44-79°	0.3	-0.21	0.53*	0.21	0.18	0.36	-0.09	-0.31	0.42	0.35	0.13	0.56
% NR >87°	-0.33	0.21	-0.24	-0.2	-0.3	-0.02	0.06	0.14	-0.1	-0.4	-0.08	-0.17
% PR <37°	0.55*	0.2	0.24	0.45*	0.54*	0.2	0.14	0.18	0.21	0.44*	0.4	0.02
% PR <56°	-0.34	0.07	-0.29	-0.28	-0.32	-0.23	0.02	0.14	-0.23	-0.3	-0.07	-0.14
% PR 44-56°	0.14	0.18	-0.1	0.57**	0.19	-0.23	0.19	0.08	-0.11	-0.12	0.24	-0.41
% PR 63-79°	-0.11	-0.02	0.25	0	0.05	0.28	0.07	0.21	0.22	0.04	0.22	0.13
% PR 44-79°	0.03	0.15	0.13	0.52*	0.21	0.04	0.24	0.27	0.1	-0.07	0.41	-0.29
% PR >87°	-0.34	-0.24	-0.25	-0.69***	-0.49*	-0.14	-0.28	-0.33	-0.2	-0.19	-0.58**	0.24
total number of PR	0.06	-0.01	0.14	-0.16	0.21	-0.03	0.11	0.17	0.09	0.25	0.16	-0.35
total number of NR	-0.02	-0.21	0	-0.31	-0.08	-0.09	-0.13	-0.23	-0.02	0.13	0.03	-0.17

Field derived traits (from Rich et al., 2016)	2012 Maximum depth	2013 Maximum depth	2013 Maximum depth	2012 90th percentile maximum depth	2013 90th percentile maximum depth	2013 90th percentile maximum depth	2012 Root penetration rate	2013 Root penetration rate	2013 Root penetration rate	2012 Total root length in core	2013 Total root length in core	2013 Total root length in core
Plot type	hill	hill	4m^2	hill	hill	4m^2	hill	hill	4m^2	hill	hill	4m^2
Paper root angle screen												
1st PS pair angle	0.0976	0.7639	0.3552	0.651	0.1787	0.3263	0.9414	0.8075	0.2436	0.5517	0.4106	0.282
2nd PS pair angle	0.516	0.0837	0.295	0.0077**	0.167	0.1971	0.0486*	0.1162	0.2343	0.4593	0.2611	0.9371
Agar root angle screen												
1st PS pair angle	0.1558	0.0959	0.5894	0.3262	0.0739	0.2318	0.1062	0.2322	0.2965	0.1859	0.5838	0.5844
Soil root angle screen												
% NR <37°	0.2305	0.6046	0.0003***	0.786	0.4879	0.0034**	0.4328	0.9544	0.0003***	0.3593	0.4	0.2127
% NR <56°	0.1254	0.5206	0.1974	0.4517	0.1871	0.379	0.7461	0.3245	0.3198	0.1145	0.7177	0.7929
% NR 44-56°	0.1611	0.2656	0.0359*	0.4999	0.4919	0.0781	0.4506	0.1726	0.1054	0.3449	0.716	0.0107*
% NR 63-79°	0.8007	0.9201	0.2124	0.5016	0.6796	0.8391	0.6034	0.7287	0.3237	0.1242	0.6304	0.9418
% NR 44-79°	0.2016	0.3855	0.0163*	0.3745	0.4412	0.1238	0.7101	0.193	0.0655	0.1247	0.5936	0.0572
% NR >87°	0.1551	0.3854	0.3171	0.4054	0.1982	0.9208	0.7957	0.5808	0.661	0.08	0.7484	0.587
% PR <37°	0.0118*	0.3969	0.302	0.0487*	0.0133*	0.4072	0.5661	0.4571	0.3688	0.0499*	0.0813	0.9584
% PR <56°	0.1393	0.7776	0.222	0.2242	0.1677	0.3274	0.9174	0.5721	0.3297	0.2042	0.7642	0.6565
% PR 44-56°	0.5542	0.4484	0.6834	0.0093**	0.4324	0.3241	0.4128	0.7434	0.657	0.6278	0.3024	0.1858
% PR 63-79°	0.6368	0.9398	0.2927	0.9955	0.8434	0.2238	0.7617	0.3864	0.342	0.8808	0.3574	0.6937
% PR 44-79°	0.8932	0.5236	0.5874	0.0182*	0.3672	0.875	0.2996	0.2572	0.672	0.7546	0.0695	0.3634
% PR >87°	0.1431	0.3035	0.2964	0.0007***	0.0288*	0.5502	0.2248	0.1746	0.387	0.4258	0.0078**	0.4553
total number of PR	0.7872	0.9709	0.5635	0.5039	0.3858	0.8968	0.6308	0.4764	0.7078	0.2953	0.4904	0.2709
total number of NR	0.937	0.3853	0.9951	0.189	0.7408	0.7056	0.5893	0.3382	0.9337	0.5919	0.909	0.5994
